# The Successful Use of Inhaled Nitric Oxide in the Management of Severe Hepatopulmonary Syndrome after Orthotopic Liver Transplantation

**DOI:** 10.1155/2014/415109

**Published:** 2014-04-03

**Authors:** Joshua Santos, Philip Young, Igor Barjaktarevic, Catherine Lazar, Irawan Susanto, Tisha Wang

**Affiliations:** ^1^Department of Internal Medicine, David Geffen School of Medicine, the University of California Los Angeles, UCLA Med-Admin, Box 951736, 37120 CHS, Los Angeles, CA 90095-1736, USA; ^2^Division of Pulmonary, Critical Care, and Sleep Medicine, David Geffen School of Medicine, the University of California Los Angeles, 10833 Le Conte Avenue, Room 37-131 CHS, Los Angeles, CA 90095-1690, USA

## Abstract

Hepatopulmonary syndrome (HPS) is characterized by pulmonary vasodilation and subsequent hypoxemia in the setting of hepatic dysfunction. There is currently no pharmacologic intervention that has been shown to significantly affect outcomes and liver transplantation remains the mainstay of therapy. Unfortunately, patients undergoing liver transplantation are at high risk of significant hypoxemia and mortality in the early postoperative period. In the following case series, we present two cases of patients with severe HPS who underwent liver transplantation and experienced marked hypoxemia in the early postoperative period. In both cases, we successfully treated the patients with inhaled nitric oxide for their severe refractory life-threatening hypoxemia which led to immediate and dramatic improvements in their oxygenation. Although the use of inhaled nitric oxide in patients with HPS has been sporadically reported in pediatric literature and in animal studies, to our knowledge, our cases are the first recorded in adult patients.

## 1. Introduction

Hepatopulmonary syndrome (HPS) represents one of the major causes of hypoxia and dyspnea among patients with end-stage liver disease. It is characterized by the triad of hypoxemia with an abnormal alveolar-arterial (A-a) gradient, presence of intrapulmonary shunting, and chronic liver disease [1]. The frequency of this disorder among patients with cirrhosis varies from 4% to 47% and may be overall underdiagnosed due to other comorbidities and pulmonary complications of liver disease that mask its presence [1]. HPS significantly influences both functional status and survival among this population of patients [1, 2]. Multiple medications have been tested for HPS with no significant improvement in oxygenation, and supportive therapy with supplemental oxygen and liver transplantation remain the only two therapies with proven benefit [3]. Unfortunately, liver transplantation itself carries a high risk of morbidity and mortality in the perioperative and postoperative period. Patients with HPS often suffer from persistent hypoxemia and are at increased risk for prolonged mechanical ventilation with a longer ICU length of stay compared to other liver transplant recipients [4, 5].

In the following case series, we present two cases of patients with severe HPS who underwent liver transplantation and experienced marked hypoxemia in the early postoperative period. In both cases, we were successful in bridging these patients with severe refractory life-threatening hypoxemia with inhaled nitric oxide which led to immediate and dramatic improvements in their oxygenation. Although there have been some reported cases in the pediatric literature of successful use of inhaled nitric oxide in patients with HPS [6, 7], to our knowledge, our cases are the first recorded in adults.

## 2. Case Presentation

### 2.1. Case 1

A 64-year-old Hispanic male with alcoholic cirrhosis presented for evaluation of hypoxia. An arterial blood gas (ABG) confirmed hypoxemia with a partial pressure of oxygen in arterial blood (PaO_2_) of 58 mm Hg and a transthoracic echocardiogram revealed a right to left shunt consistent with a diagnosis of hepatopulmonary syndrome (HPS). Further testing showed a 36% right to left shunt on a whole body radiolabeled macroaggregated albumin scan, and contrast chest CT demonstrated minimal peripheral reticular ground glass opacities consistent with mild pulmonary fibrosis without evidence of discrete arteriovenous malformations. Pulmonary function tests (PFTs) revealed severe diffusion impairment without restriction.

The patient was initiated on continuous supplemental oxygen at 2 liters per minute and was listed for orthotopic liver transplant (OLT) with a Model for End Stage Liver Disease (MELD) exception for HPS [8]. Over the next year, his oxygen requirements increased to 5 L with a PaO_2_ of 49 mm Hg on room air. No change was noted in his PFTs or chest CT.

Fourteen months after his initial presentation, the patient underwent OLT without any intra-operative complications. He was extubated on post-operative day (POD) 3 and discharged home on POD 10 to a respiratory rehabilitation facility on 5 L of supplemental oxygen at rest. Three days after the discharge, he was readmitted to the hospital with dyspnea and profound desaturation into the 50 s on pulse oximetry. He was emergently intubated and had persistent oxygen saturations in the 50 s with nadir PaO_2_ of 26 mm Hg on the mechanical ventilator with FiO_2_ 1.0. Given his severe refractory hypoxemia and minimal improvement with supine positioning, he was placed on inhaled nitric oxide (NO) at 20 parts per million (ppm) with improvement of oxygenation first hour of NO treatment. A repeat ABG on FiO_2_ 0.8 revealed a PaO_2_ of 113 mm Hg. Chest CT was unrevealing for thromboembolic disease or any other secondary cause to explain his acutely worsening hypoxemia. The mild pulmonary fibrosis appeared grossly unchanged. Over the next several days, the patient was slowly weaned from the ventilator and the inhaled NO. He was started on garlic and pentoxifylline. He was successfully extubated on hospital day 4 to high flow nasal cannula with FiO_2_ of 0.6 and again discharged to a respiratory rehabilitation facility on hospital day 12.

Two months after OLT, the patient was weaned off of oxygen, garlic, and pentoxifylline. His dyspnea markedly improved and his saturations were noted to be 95% on room air. He continues to do well with a functioning liver and no recurrent hypoxia or pulmonary complaints.

### 2.2. Case 2

A 55-year-old Caucasian male with cirrhosis secondary to hemochromatosis and heavy alcohol use presented with dyspnea on exertion and a resting oxygen saturation of 87%. ABG revealed a PaO_2_ of 55 mm Hg on room air with a shunt fraction of 20% while on 100% oxygen. A transthoracic echocardiogram with contrast demonstrated an extracardiac shunt consistent with HPS. A CT scan of the chest with contrast demonstrated mild interstitial lung disease, distributed primarily in the periphery and lower lung bases without evidence of arteriovenous malformations. PFTs revealed a mild restrictive ventilatory defect with severe diffusion impairment. A work-up for secondary causes of interstitial lung disease was negative.

**Figure 1:**
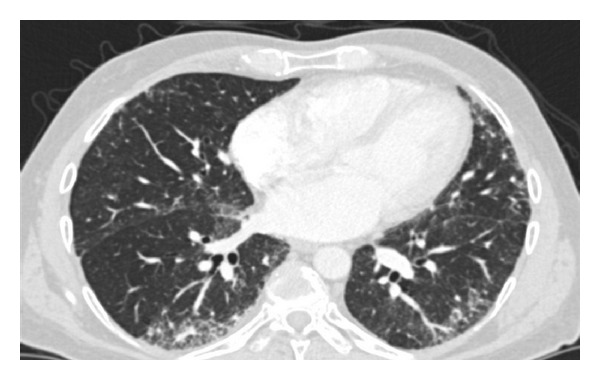
Chest CTA showing subpleural pulmonary fibrosis, inter- and intralobular septal thickening, architectural distortion with traction bronchiectasis and bronchiolectasis, and distortion of interfaces as well as diffuse prominence of the segmental and subsegmental pulmonary arteries with extension of vessels to the lung periphery.

The patient was placed on home oxygen and listed for OLT with a MELD exception for HPS. Six months after his initial evaluation, his PaO_2_ had declined to 46 mm Hg on room air and his oxygen requirement increased to 4 L of oxygen. He was treated with garlic 2 grams daily and N-acetylcysteine for HPS and pulmonary fibrosis, respectively. His hypoxemia continued to worsen with a PaO_2_ of 34 mm Hg on room air, requiring 10 L of supplemental oxygen with exertion. PFTs also demonstrated worsening restriction and diffusion impairment (DLCO 27% of predicted). A repeat CT scan (Figure 1) showed worsening of his basilar and subpleural pulmonary fibrosis. Given his profound orthodeoxia and the observation that his oxygen requirements remained significantly out of proportion to his mild to moderate pulmonary fibrosis, it was felt that the majority of his hypoxemia was still related to his HPS. Nine months later, the patient underwent an OLT. On POD 1, his oxygenation worsened with a PaO_2_ of 30 mm Hg while on FiO_2_ 1.0 on the mechanical ventilator. A trial of increased positive-end expiratory pressure (PEEP) worsened his hypoxemia. The patient was initiated on inhaled NO at 20 ppm and within one hour, his PaO_2_ rose from 50 to 154 mm Hg while on a FiO_2_ of 1.0. In the ensuing 24 hours, his PaO_2_ improved to 93 mm Hg on FiO_2_ of 0.45. The NO was weaned off on POD 4 and he was subsequently extubated to 4 L of oxygen via nasal cannula. By POD 16, he was on room air at rest and eventually discharged home on POD 25 with 1 L of oxygen to use as needed with exertion.

Four weeks after discharge, he was seen in pulmonary clinic and reported dramatic improvement in his symptoms of dyspnea. His PaO_2_ improved to 79 mm Hg on room air, and he continues to do well with improvement of his PFTs and stability of his CT imaging findings.

## 3. Discussion

The two most commonly accepted therapeutic approaches for HPS are supportive oxygen therapy and liver transplantation, which is the only curative modality. Numerous case reports have described significant hypoxemia in HPS patients undergoing liver transplantation during the perioperative period—similar to our aforementioned patients—that leads to severe morbidity and mortality [5, 9, 10]. Compared to one-year overall survival of approximately 90% for non-HPS OLT patients, the one-year postoperative survival for patients with HPS is 71% and the majority of lethal outcomes within 10 weeks post-OLT are the result of respiratory complications [5]. Specifically, severe HPS—defined as a PaO_2_ of <50 mm Hg or a shunt fraction of >20% on a macroaggregated albumin study—has been found to significantly increase mortality, with or without OLT [11]. These findings suggest that the presence of HPS is an independent predictor of increased mortality after liver transplantation, particularly in the setting of severe hypoxemia.

Unfortunately, there has been no successful pharmacologic therapeutic intervention which has changed the clinical course, quality of life, or mortality in patients with HPS. Various medical approaches such as garlic [12] or pentoxifylline [13] therapy have been used with very modest success. The lack of successful pharmacologic agents in the management of HPS is partially due to the uncertainty that surrounds the pathogenesis behind this disorder. An imbalance in favor of vasodilators over vasoconstricting substances has been described implicating nitric oxide (NO) as a potential causal vasoactive substance. This hypothesis has been further supported by the fact that both endothelial nitric oxide synthase (eNOS) and inducible nitric oxide synthase (iNOS) show increased expression in the pulmonary endothelium of HPS patients [3, 14]. In addition, exhaled NO in the breath of cirrhotic patients is increased in HPS with the concentration normalizing after liver transplantation [15]. However, nitric oxide antagonists such as methylene blue and NG-nitro-l-arginine-methyl ester (l-NAME) have been evaluated in the treatment of HPS with little to no success [16, 17].

The treatment of hypoxia and respiratory failure due to HPS with nitric oxide—conceptually an opposite approach—has been reported in animal models [18]. There have also been case reports in the pediatric population demonstrating similar findings with the use of inhaled NO in patients with HPS, though none to our knowledge in adults [6, 7, 19].

As described above, both of our patients with severe HPS experienced marked hypoxemia in the early postoperative period. In spite of a thorough diagnostic work-up, no other significant modifiable etiology was found for these patients' profound hypoxemia and we concluded that the worsening ventilation/perfusion (V/Q) mismatch from underlying HPS was the primary cause with a few other minor factors possibly contributing, such as volume shifts, atelectasis, and mild ILD. In both cases, their severe refractory life-threatening hypoxemia dramatically improved after inhaled NO treatment and helped liberate them from the ventilator. With their functioning liver grafts, both patients were able to wean off supplemental oxygen shortly after OLT. The exact mechanism is unclear, but we speculate that inhaled NO led to more uniform vasodilation of the pulmonary vasculature, thereby redistributing blood flow to apical and mid-lung fields resulting in an improvement in the V/Q mismatch. A similar mechanism has been proposed for garlic therapy which does have a modest effect on oxygenation in patients with HPS [12].

Though it is difficult to make definitive conclusions based on two isolated cases, inhaled NO could prove beneficial in a subset of patients with severe HPS, either as a bridge to OLT or as a salvage therapy in the peri- and post-operative period to allow for the successful transplantation of patients with HPS and associated severe hypoxemia. Improving oxygenation in the postoperative period could lead to earlier weaning from mechanical ventilation and potentially prevent nonpulmonary complications as well. Further studies are clearly warranted to investigate the effect of inhaled NO in the setting of HPS and our center is currently embarking on one of these projects.

## Abbreviations

ABG: Arterial blood gas
ARDS: Adult respiratory distress syndrome
HPS: Hepatopulmonary syndrome
DLCO: Diffusion capacity
ICU: Intensive care unit
MELD: Model for end-stage liver disease
NO: Nitric oxide
OLT: Orthotopic liver transplantation
PaO_2_: Partial pressure of oxygen in arterial blood
PFT: Pulmonary function test
POD: Postoperative day
V/Q: Ventilation/Perfusion.
